# Perceptions of Control Influence Feelings of Boredom

**DOI:** 10.3389/fpsyg.2021.687623

**Published:** 2021-07-09

**Authors:** Andriy A. Struk, Abigail A. Scholer, James Danckert

**Affiliations:** Department of Psychology, University of Waterloo, Waterloo, ON, Canada

**Keywords:** boredom, perceived control, challenge, engagement, frustration

## Abstract

Conditions of low and high perceived control often lead to boredom, albeit for different reasons. Whereas, high perceived control may be experienced as boring because the situation lacks challenge, low perceived control may be experienced as boring because the situation precludes effective engagement. In two experiments we test this proposed quadratic relationship. In the first experiment we had participants play different versions of the children's game “rock-paper-scissors” in which they arbitrarily won (intended to maximize feelings of control) or lost (to induce feelings of low control). Despite having only dichotomous conditions, participants reported experiencing a broad range of levels of perceived control. Consistent with our predictions, boredom was highest at low and high levels of perceived control (i.e., a quadratic relation between perceived control and felt boredom). Experiment 2 tested the notion that the mere prospect of gaining control may mitigate boredom. Participants given to believe (erroneously) that they could gain control over the game of rock, paper, scissors were less bored than those who believed there was no possibility of winning at greater than chance levels. This suggests that beliefs concerning prospective control, rather than a given level of perceived control *per se*, may predict engagement and boredom.

## Introduction

Boredom is ubiquitous and prevalent, experienced about one third of the time by high school and university students, and reported as one of the most prevalent negative affective experiences in adults (Larson and Richards, 1991; Goetz et al., 2013; Chin et al., 2016). Furthermore, there is a recent suggestion that in adolescents boredom is on the rise (Weybright et al., 2020). The tendency to experience boredom more frequently and intensely—so-called boredom proneness (Tam et al., 2021)—is associated with a raft of negative outcomes, including substance abuse (Wiesner et al., 2005; Amos et al., 2006; LePera, 2011; German and Latkin, 2012), depression (Goldberg et al., 2011), and poor academic achievement (O'Hanlon, 1981; Maroldo, 1986). The in-the-moment feeling of boredom—state boredom—is an aversive experience arising from an inability to engage in satisfying activities (Eastwood et al., 2012), a notion consistent with the finding that poor self-control is linked with increased boredom proneness (Struk et al., 2015; Isacescu et al., 2017; Mugon et al., 2020; Wolff et al., 2021).

Although boredom is aversive, it may serve a self-regulatory function (Eastwood et al., 2012; Bench and Lench, 2013, 2019; Elpidorou, 2014, 2018). Boredom arises in situations considered deficient in meaning, challenge or interest, and motivates actions to address these deficiencies (Sansone et al., 1992; Fahlman et al., 2009; Smith et al., 2009; van Tilburg and Igou, 2011, 2012; Mercer-Lynn et al., 2014; Tam et al., 2021). In other words, boredom functions as a prompt to escape a boring situation and explore alternative activities (Bench and Lench, 2013, 2019; Goetz et al., 2013; Elpidorou, 2014, 2018).

The *need for control*—feeling effective and competent in the pursuit of goals—is considered a core human motive (Deci and Ryan, 1985; Fiske, 2009; Higgins, 2014). Feelings of control may arise from congruence between one's behavior and desired outcomes (Fiske, 2009). Even when belief in control is illusory (Taylor and Brown, 1988; Higgins, 2014), it is the *perception* of control that is critical for well-being (e.g., Pittman and Pittman, 1979; Taylor and Brown, 1988). Perceiving that one is in control makes individuals feel that the world is predictable and knowable and that they can navigate it effectively (Bandura, 1977; Ajzen, 1991).

Given the role of perceptions of control in engagement, it is not surprising that research has posited a link between boredom and perceived control. Students reporting a lack of perceived control were more prone to boredom (Watt and Vodanovich, 1999; Pekrun et al., 2010). Likewise, students found learning activities to be most boring when they had low levels of autonomy (Dicintio and Gee, 1999; Nett et al., 2010; Pekrun et al., 2010). Similarly, students who equated schooling with boredom also reported a lack of perceived control and choice in their learning experience (Kanevsky and Keighley, 2003; see also Troutwine and O'Neal, 1981 for a demonstration of the influence of choice on time spent on a boring task). These findings suggest that low levels of perceived control often lead to boredom.

However, experiencing *high* levels of perceived control may also be a precursor for boredom when individuals no longer feel challenged. Indeed, students report that a lack of *challenge* contributed to boredom (Kanevsky and Keighley, 2003; see also van Tilburg and Igou, 2012). Consistent with this finding, researchers have developed methodologies for job rotation under the assumption that skill mastery leads to boredom (Azizi et al., 2010). These findings suggest an optimal “Goldilocks” level of perceived control over a given task that is ideal for engagement; not so little to preclude effective engagement, and not so much as to promote monotony. In one sense, this casts boredom in the context of information processing (Klapp, 1986). Constantly changing environs make it difficult if not impossible to differentiate signal from noise. At the other extreme, a monotonous task involves a high degree of redundancy, with little to no new information to be gleaned (Klapp, 1986). These notions evoke theories of achievement motivation, which suggests people are motivated to engage in activities with sufficiently high likelihood of success, but not so high as to be absent of any challenge (e.g., McClelland, 1951; Atkinson, 1957). This is also consistent with the Control-Value theory of achievement emotions, which outlines how value and control interact to enable distinct affective experiences (Pekrun, 2006). For boredom, it is predicted that conditions of high *or* low perceived control will lead to boredom, by reducing the incentive value, or informational gain, of an activity. Prior work examining the relationship between perceived control and boredom has largely focused on trait boredom, or did not directly assess the relation between perceived control and boredom (Roth and Kubal, 1975; Troutwine and O'Neal, 1981; Dicintio and Gee, 1999; Watt and Vodanovich, 1999; Kanevsky and Keighley, 2003; Pekrun et al., 2010). Here in two experiments, we test the hypothesis that perceived control exhibits a curvilinear relationship with boredom, such that both low and high levels of perceived control will be associated with elevated boredom. This hypothesis is based on the theoretical accounts of boredom discussed above and rests heavily on Pekrun's Control-Value theory of academic achievement (2006). This theory proposes that boredom arises when an activity is appraised as *either* high or low in levels of perceived control *and* low in value (e.g., the task appears irrelevant to one's goals).

## Experiment 1

Experiment 1 assessed the relation between perceived control and boredom. To manipulate perceived control we used a computerized version of the children's game of “rock-paper-scissors” (RPS) in which individuals played against a computer opponent with differing win rates. This is a task we have used elsewhere to examine the capacity to learn and exploit an opponent's strategy (Danckert et al., 2012). In that study, healthy individuals were indeed able to exploit an opponent's play strategy. We capitalized on this fact by assuming that telling participants that the opponent would play an exploitable strategy would lead to concerted attempts to discover and exploit that strategy. Given that our instructions were deceptive (i.e., the computer did not adopt an exploitable strategy—see below) we assumed that this would be an effective way to manipulate perceived levels of control (a manipulation we ultimately checked *via* self-reports). In other words, participants were made to believe there was an exploitable strategy to discover and in one condition they would never successfully discover it (100% losses condition) while in the other they would assume they had rapidly figured it out (100% wins conditions). Such circumstances should in turn lead to low and high levels of perceived control. Given past research on the illusion of control, we felt justified in adopting this manipulation. That is, humans do typically pursue circumstance than enable them to establish control and often claim a sense of control over events that are objectively random (Thompson, 1999).

Participants were randomly assigned to one of two conditions; in the first, regardless of play choices, participants won 100% of the time—a condition which was assumed to provoke high levels of perceived control. In the second group, regardless of play choices participants lost games 100% of the time—a condition intended to evoke low perceived control. In other words, when individuals won frequently we assumed they would attribute this to the fact that they had “figured out” the computer's strategy, an illusory sense of control. In contrast, when individuals always lost they should experience this as a failure to figure out their opponent's strategy, thereby creating the sense of low perceived control.

To explore how behavior was affected by the expected relation between perceived control and boredom, we calculated indicators of (dis)engagement that assessed how long participants deliberated on each trial and the extent to which they explored different strategies within the game. We predicted that individuals would display greater task engagement in the 100% losses (vs. 100% wins) condition, characterized by broader exploration of strategy space and longer deliberation times while choosing their playing strategy. This hypothesis was based on the assumption that in the 100% losses condition participants would persist in the attempt to discern their opponent's strategy despite constant failure (see below for details on these metrics).

In addition to state boredom, we included probes of challenge, value, and frustration for three reasons; first, we wanted to examine the robustness of the relationship between perceived control and boredom (e.g., is perceived control or challenge/value a better predictor of boredom?). Second, because our manipulation was tied to different win rates, it is possible that participants would place different values on the task, and that such variation in assigned value may account for differences in boredom. Thus, it was important both to examine whether our manipulation affected perceptions of value and to demonstrate that the relationship between perceived control and boredom remained when controlling for value. Third, we wanted to explore whether the phenomenological experience of the two conditions could be further differentiated by frustration. That is, frustration may be presumed to be higher in the 100% losses condition given that any efforts to figure out their opponent's strategy were ultimately (by design) fruitless.

### Method

#### Participants

One hundred and ninety-four undergraduates [137 females, mean age = 20.49 (2.56) years; 35.4% East Asian, 31.2% Caucasian, 21.2% “other” Asian, and 12.2% “other”] from the University of Waterloo participated in exchange for course credit. Participants were randomly assigned to one of two experimental conditions. Data was collected during Spring and Fall terms of 2014. We decided, a priori, to collect as many participants as possible over the two academic terms. Data was not analyzed until the entire sample was collected. This study was approved by the University of Waterloo, Office of Research Ethics and participants gave informed consent prior to participating.

#### Apparatus

A computerized version of the RPS game was programmed using python 2.7 and a pygame library. The game was displayed on 16″ CRT monitor with a resolution of 1024 × 768. Participants sat ~50 cm from the monitor and used a mouse to make responses. Computer choices and participants' responses appeared in 210 × 210 pixel boxes.

#### Procedure

**Figure 1:**
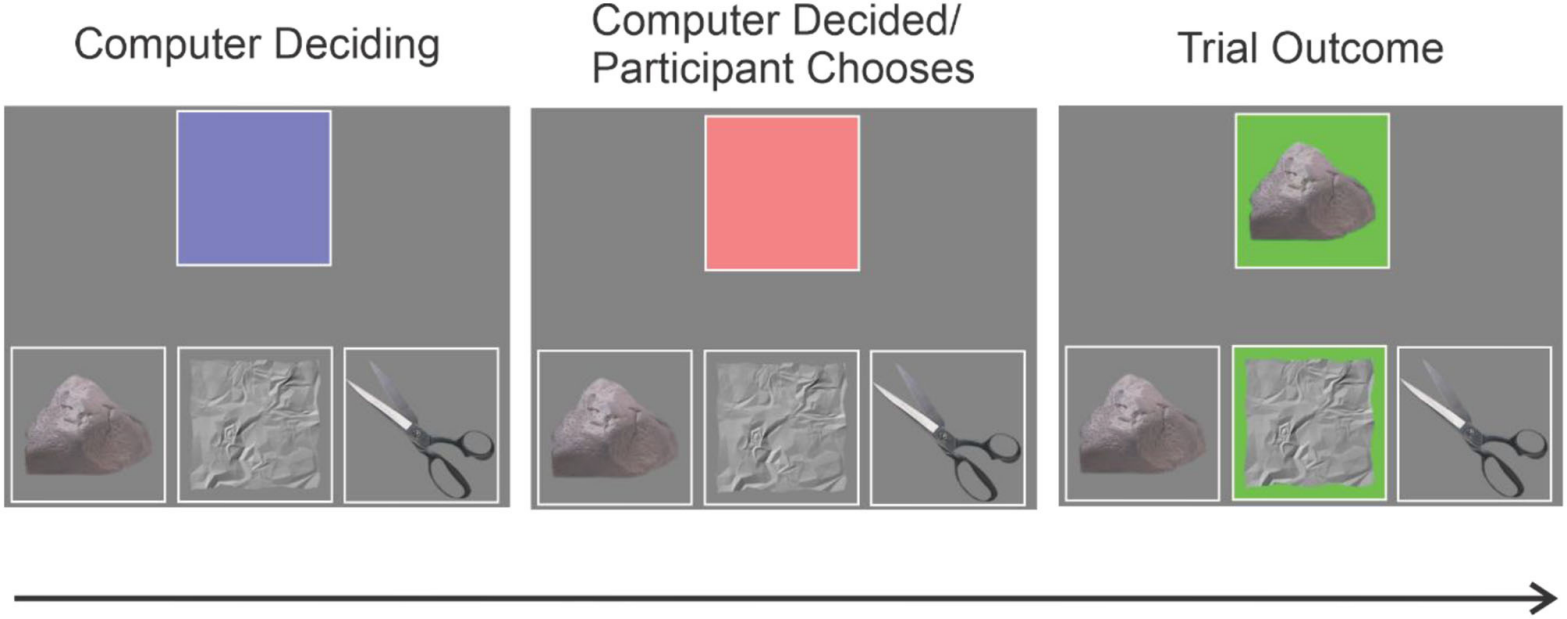
Schematic of a single trial in the “rock, paper, scissors” game. The top square in the center of the screen represented the computer opponent. While “choosing” an option this was colored blue **(left)**. When the computer had chosen the square changed colors to red indicating that the participant could now choose **(middle)**. When the participant chose one option, the computer's choice and the trial outcome were revealed, with a win for the participant indicated by a green background and a loss by a red background (given our task design no ties were possible in this version of the game).

On each trial participants viewed a blue square in the upper half of the screen that represented the computer's choice. Blue indicated a choice was pending and after 500 ms the square turned red, indicating a choice had been made and participants could now play their response. The three response options (e.g., rock, paper, scissors) were always visible on the lower half of the screen (Figure 1). Participants responded by clicking on their choice, at which point the computer's choice was revealed, and both squares were highlighted in red or green, depending on whether the participant lost or won (ties were not possible). Participants were told the computer played an exploitable strategy and were instructed to attempt to discover that strategy and exploit it to maximize their wins. There was in fact, no exploitable strategy. Instead, participants were randomly assigned to either the 100% wins or high perceived control condition, in which participants won all trials, or the 100% losses or low perceived control condition, in which participants lost all trials. These contingencies were unknown to the participant. Each participant played 20 hands of RPS, after which they answered probes concerning boredom, perceived control, frustration, challenge, and the level of value they assigned to the task (Table 1). Participants responded to probes on a 100 point visual slider scale with anchors of “not at all” to “extremely.”

**Table 1:** Study variables and probe questions.

**Variable**	**Question**
Perceived control	“To what extent do you feel in control in the task?”
Boredom	“How bored are you?”
Frustration	“How frustrated are you?”
Value	“How much do you care about winning?”
Challenge	“How challenging is this task?”

#### Data Analysis

All data analyses were conducted in R (R Core Team, 2015). To index task engagement we computed a measure of strategy complexity (how simple or elaborate are participants' sequences of choices?), strategy variability (do participants stick with a strategy?), and response time (RT; the time to respond after the computer has chosen). Examining strategy use is not simple in a “rigged” game. It makes little sense to explore the adoption of win-stay/lose-shift strategies—common in decision making research—as each condition has 100% wins or losses. Representing simple proportions of plays (Danckert et al., 2012) also fails to capture the type or variability of adopted strategies. To address these limitations, we first examined the frequency of single choice plays—how often did an individual play the same choice played on trial *n*−1 This was calculated as the proportion of trials on which participants made a response that was identical to that made on the previous trial (labeled R1 here). To examine more complex strategies, we looked at 2 and 3 trial sequences. A two-trial sequence reflects simple alternation (e.g., R-P-R-P…). Three-trial sequences are more complex with a variety of permutations that represent a consistent, strategic approach. We examined the proportion of trials on which alternation (R2) or sequential (R3) strategies were employed and combined those approaches as “complex” strategies.

These strategies lie on a continuum. On one end are individuals who play the same response repeatedly; this type of regularity may indicate a lack of effort perhaps indicative of giving up. On the other end are individuals with a more variegated, yet regular, game play that represents an established strategy (e.g., consistently playing R-R-P as a triplet). Both ends of the continuum reflect lower levels of exploration of strategy space, while the middle represents an active exploration of the opponent's strategy. To capture whether individuals favored simple or complex strategies we computed a Strategy Complexity (SC) metric. To do this we subtracted the average proportion of R1 plays from the proportion of R2 and R3 plays for each individual. A negative SC indicates adoption of simple strategies, whereas a positive SC indicates the adoption of complex strategies.

Next, we examined Strategy Variability (SV) to capture how consistently any given strategy was adopted. To do this we computed Shannon's entropy (Shannon, 1948), based on the proportion of all strategies. This represents the degree of disorder or uncertainty within a set of events (in our case strategy types in the RPS game). Entropy is zero when we are completely certain of the outcome; for instance, if an individual alternates between two options (e.g., R-P-R-P, etc.) for the entire experiment. On the other extreme, maximum entropy is two, which occurs when an individual engages in every type of strategy play equally. The benefit of this measure is that if one strategy is used frequently (regardless of which), SV will yield smaller values indicating greater adherence to a given strategy.

To visualize differences in task engagement, we can represent the strategy space as a 2D vector: with three possible strategies [simple (R1), complex (R2 and R3), and “other”] there are 2 degrees of freedom, thus, the proportion of trials on which some strategy other than a simple or complex strategy was adopted, can be inferred from knowing the proportions of simple and complex strategies. Finally, given that many of these states are visited multiple times between subjects, we applied a density function to reveal the distribution of strategies across all participants. Higher density indicates that one type of strategy predominates, indicating low SV—a more diffuse density plot indicates greater exploration of strategy space and thus a high SV. Higher density in the top left corner indicates preference for a simple strategy or low SC, while higher density in the bottom right corner indicates a preference for a complex strategy or high SC.

### Results

Gender proportions did not differ across conditions $X_{\left(1\right)}^{2}$ = 1.24, *p* = 0.27. No gender differences were observed across all study variables with the exception of mean time to respond, whereby females (mean = 1.14 s) were significantly slower than males (mean = 0.95 s; *t* = 4.08, *p* < 0.0005, *Cohen's d* = 0.66).

As a manipulation check we explored differences between ratings of perceived control across the two groups. As predicted, perceived control in the 100% wins condition was significantly higher than in the 100% losses condition (*t* = 14.76, *p* < 0.0001, *Cohen's d* = 2.12). Next, we explored the effect of our manipulation on boredom and frustration. As predicted, the 100% losses condition was perceived as more frustrating (*t* = 10.74, *p* < 0.0001, *Cohen's d* = −1.54), while the 100% wins condition was rated as more boring (*t* = 3.84, *p* = 0.0002, *Cohen's d* = 0.55). There was no difference in perceived value (*t* = 0.053, *p* = 0.956, *Cohen's d* = 0.01). However, the 100% losses condition was seen as more challenging (*t* = 12.82, *p* < 0.0001, *Cohen's d* = −1.84).

Next, we explored zero-order correlations between all study variables in both groups collapsed (correlations collapsed across condition are shown in Table 2A; High Perceived Control condition correlations are shown in Table 2B, with Low Perceived Control condition correlations in Table 2C). Boredom was positively associated with perceived control (*r* = 0.26, *p* < 0.001), and negatively with challenge (*r* = −0.21, *p* < 0.01), value (*r* = −0.21, *p* < 0.01) and frustration (*r* = −0.16, *p* < 0.05). Perceived control was positively associated with strategy complexity (*r* = 0.26, *p* < 0.001), and negatively with frustration (*r* = −0.55, *p* < 0.001), and strategy variability (*r* = −0.44, *p* < 0.001). There was no association between perceived control and value (*r* = 0.12, *p* = 0.11).

**TABLE 2A:** Zero-order correlations of all study variables and their significance levels (Study 1).

	**1**	**2**	**3**	**4**	**5**	**6**	**7**
1. Perceived control							
2. Bored	0.26^***^						
3. Frustration	−0.52^***^	−0.16^*^					
4. Challenge	−0.55^***^	−0.21^**^	0.54^***^				
5. Value	0.09	−0.21^**^	0.33^***^	0.09			
6. Strategy Complexity	0.26^***^	0.03	−0.22^**^	−0.23^**^	0.13		
7. Strategy Variability	−0.44^***^	−0.14	0.34^***^	0.32^***^	−0.03	−0.18^*^	
8. Response Times	−0.04	−0.15^*^	0.15^*^	0.11	0.19^**^	0.21^**^	−0.07

* *p < 0.05*,

** *p < 0.01*,

*** *p < 0.001*.

**TABLE 2B:** Zero-order correlations of all study variables in the *High Perceived Control* condition and their significance levels.

	**1**	**2**	**3**	**4**	**5**	**6**	**7**
1. Perceived control							
2. Bored	0.30^**^						
3. Frustration	−0.11	0.02					
4. Challenge	−0.09	0.01	0.48^***^				
5. Value	0.19	−0.15	0.01	−0.15			
6. Strategy complexity	0.05	−0.11	−0.24^*^	−0.07	0.11		
7. Strategy variability	−0.35^***^	−0.05	0.24^*^	0.22^*^	−0.16	0.19	
8. Response times	0.1	−0.15	−0.04	−0.03	0.11	−0.22^*^	−0.29^**^

* *p < 0.05*,

** *p < 0.01*,

*** *p < 0.001*.

**TABLE 2C:** Zero-order correlations of all study variables in the *Low Perceived Control* condition and their significance levels.

	**1**	**2**	**3**	**4**	**5**	**6**	**7**
1. Perceived control							
2. Bored	−0.18						
3. Frustration	−0.14	−0.01					
4. Challenge	−0.12	−0.07	0.18				
5. Value	0.03	−0.29^**^	0.64^***^	0.23^*^			
6. Strategy complexity	0.01	0.03	0.11	−0.02	0.18		
7. Strategy variability	−0.03	−0.05	0.1	0.05	0.25^*^	−0.68^***^	
8. Response times	−0.06	−0.14	0.18	0.1	0.26^*^	−0.33^**^	0.19

* *p < 0.05*,

** *p < 0.01*,

*** *p < 0.001*.

**Figure 2:**
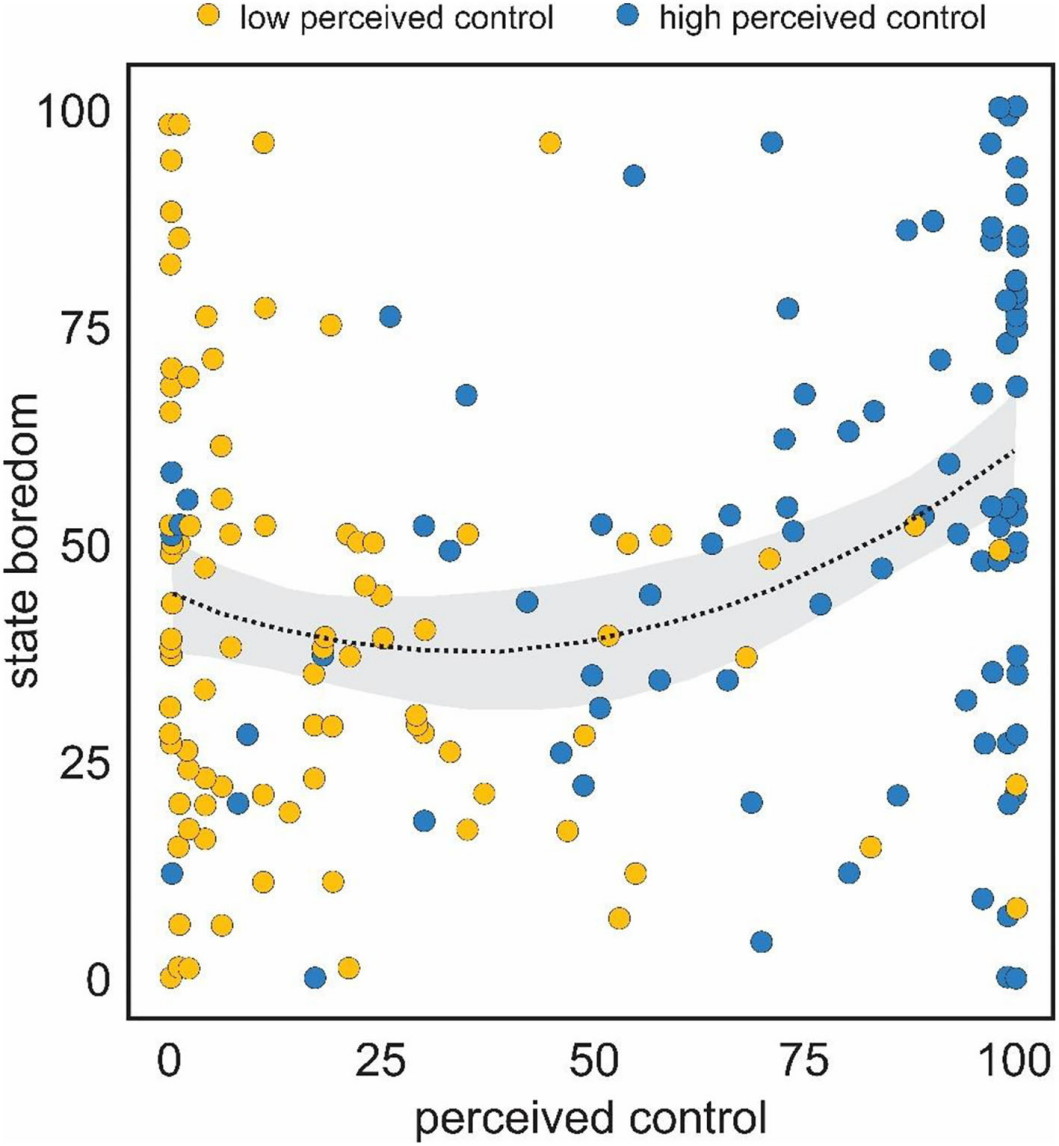
Curvilinear relationship between self-reported ratings of state boredom (y-axis) and perceived control (x-axis). Boredom was highest at the low and high ends of perceived control.

To examine whether the relation between boredom and perceived control follows a U-shaped function, we conducted a hierarchical regression. First, all variables were scaled and centered, and a new squared variable generated. Next, we tested whether second order polynomials incrementally improved model fit over first order relationships. The linear relationship between perceived control and boredom was significant [*F*_(1,192)_ = 13.9, *p* < 0.001, β = 0.26, adjusted *R*^2^ = 0.068]. Addition of the quadratic term significantly improved model fit [*F*_(1,191)_ = 7.039, SS = 6.38, *p* < 0.01]. Both the linear (β = 0.23, *p* < 0.01), and quadratic terms (β = 0.18, *p* < 0.01), were significant predictors of boredom, with an overall significant model fit [*F*_(2,190)_ = 10.86, *p* < 0.0001, adjusted *R*^2^ = 0.093]. Both low *and* high perceived control were associated with higher boredom, suggesting that the relation was curvilinear (Figure 2). No other variable demonstrated a curvilinear relationship with boredom. Furthermore, when controlling for all other subjective and behavioral measures, the curvilinear relationship between perceived control and boredom remained significant (β = 0.19, *p* < 0.01).

To explore whether perceived control and value served as independent predictors of boredom we ran an incremental regression analysis, in which value was added as a predictor to a model that had the linear and quadratic terms of perceived control as predictors of boredom. Adding value significantly improved the model fit [*F*_(1,190)_ = 11.36, *SS* = 9.78, *p* < 0.001], accounting for an additional 4.8% of variance. Although value was a significant negative predictor (β = −0.23, *p* < 0.001) of boredom, its addition did not affect the linear (β = 0.24, *p* < 0.001) or quadratic terms (β = 0.18, *p* < 0.01) of the relation between perceived control and boredom, both of which remained significant positive predictors of boredom, with an overall significant model fit [*F*_(3,189)_ = 11.50, *p* < 0.0001, adjusted *R*^2^ = 0.141].

In addition to affecting perceived control, our manipulation also influenced perceived challenge. Thus, we examined whether boredom levels were best accounted for by differences in perceived control or challenge. To do this, we tested whether perceived levels of challenge accounted for *additional* variance in reported boredom levels beyond perceived control using hierarchical regression. On its own challenge was a significant predictor of boredom [*F*_(1,192)_ = 9.0, *p* < 0.005, adjusted *R*^2^ = 0.040]. Likewise, perceived control operated as a significant predictor of boredom [*F*_(1,192)_ = 13.9, *p* < 0.001, adjusted *R*^2^ = 0.062]. Adding reported levels of challenge to a model in which only perceived control was a predictor of boredom did not improve the model fit [*F*_(1,191)_ = 1.44, *SS* = 1.389, *p* = 0.224], suggesting that challenge does not explain any *additional* variance in boredom beyond that which is explained by perceived control. In this final model, perceived control operated as a significant positive predictor (β = 0.21, *p* < 0.05), and challenge as a *non-significant* negative predictor [β = −0.10, *p* = 0.22; model fit statistics: *F*_(2,191)_ = 7.709, *p* < 0.001, adjusted *R*^2^ = 0.065]. Thus, perceived control explains the same variance in boredom as challenge, and serves as a better predictor of boredom.

**Figure 3:**
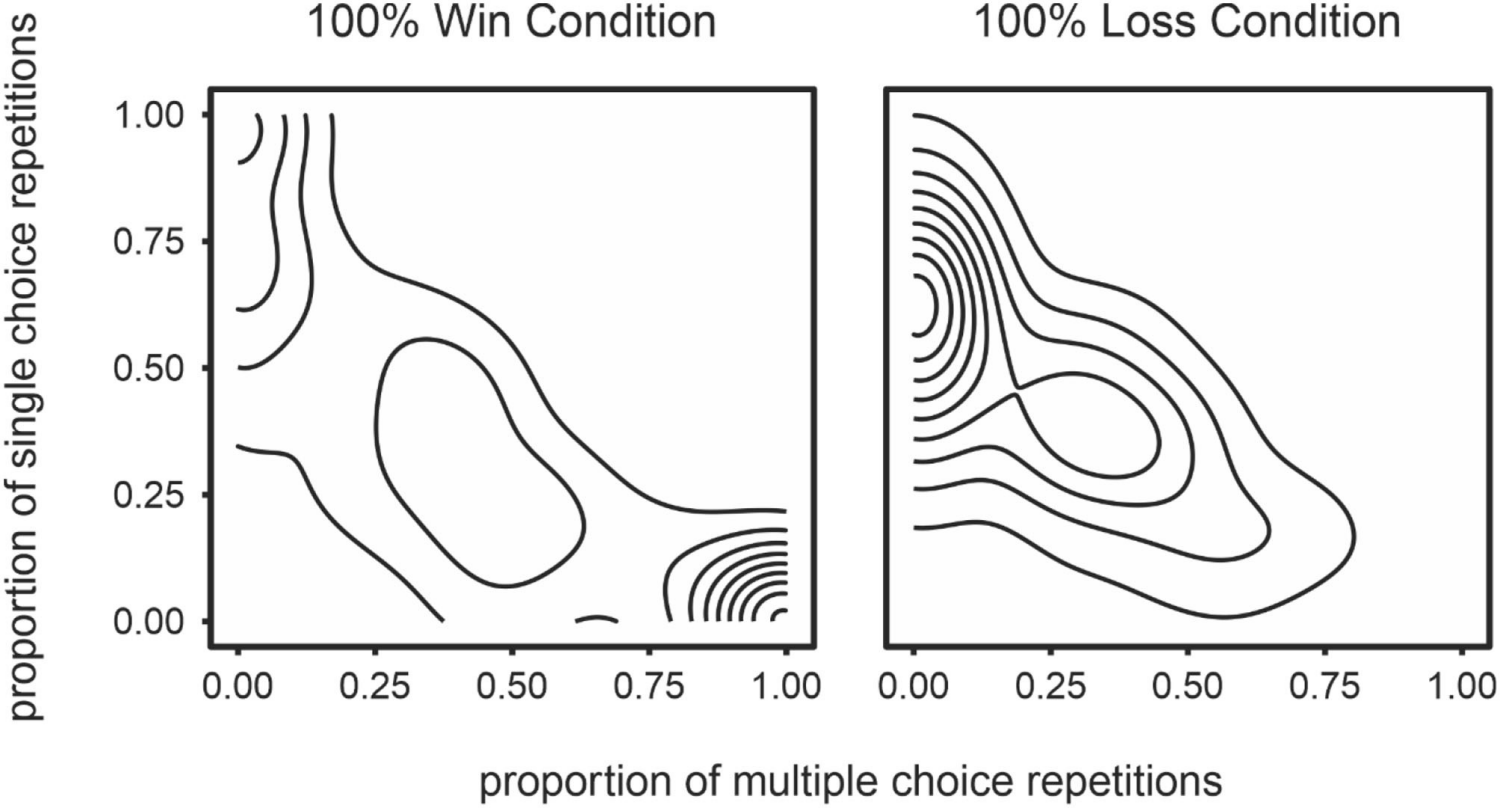
Density plots representing exploration of strategy space in the 100% win (left panel) and 100% loss (right panel) conditions. The proportion of trials on which play involved complex strategies (alternations and sequences) are presented on the x-axis and the proportion of trials where a single choice was played repeatedly is presented on the y-axis. The density plot was generated by collapsing across all participants, for each condition separately. Regions with many concentric lines indicate a high density or frequency of that particular distribution of strategies.

To test whether perceived control affects engagement, we tested differences in behavioral measures across the two groups. First, we tested the effect of our manipulation on strategy variability. As predicted, participants in the 100% losses condition exhibited higher strategy variability than those in the 100% wins condition (SV; *t* = 5.83, *p* < 0.0001, *Cohen's d* = −0.85; Table 3 and Figure 3). Next, we tested how our manipulation affected strategy complexity. Participants in the 100% losses condition exhibited less strategy complexity than those in the 100% wins condition (SC; *t* = 4.38, *p* < 0.0001, *Cohen's d* = 0.66; Table 3 and Figure 3). Contrary to our prediction, participants did not take longer to respond in the 100% losses condition (*t* = 1.08, *p* = 0.283, *Cohen's d* = −0.15).

**TABLE 3:** Descriptive statistics for all variables and effect sizes (Study 1).

		**High perceived control** ***n*** **=** **98**			**Low perceived control** ***n*** **=** **96**				
**Variable**	**Mdn**	**M**	**SD**	**Mdn**	**M**	**SD**	***t***	***p***	***d***
Control	96	77.54	30.32	6.5	18.71	24.99	14.76	<0.0001	2.12
Boredom	52.5	54.4	27.11	38.5	40.18	24.46	3.84	0.0002	0.55
Frustration	3	8.41	13.55	52.5	46.52	32.09	10.74	<0.0001	−1.54
Challenge	1	4.48	9.85	59	54.1	36.63	12.82	<0.0001	−1.84
Value	50	47.79	29.38	51	47.56	28.95	0.053	0.956	0.01
SC	0.07	0.1	0.73	−0.4	−0.29	0.42	4.38	<0.0001	0.66
SV	0.58	0.49	0.44	0.69	0.79	0.25	5.83	<0.0001	−0.85
RT	0.97	1.07	0.32	1.03	1.13	0.46	1.08	0.283	−0.15

*Mdn, median; M, mean; SD, standard deviation; d, Cohen's d; SC, strategy complexity; SV, strategy variability; RT, response time*.

### Discussion

To investigate the influence of perceived control on boredom we manipulated win rate in the children's game of rock, paper, scissors to induce either a high or low sense of control. Self-reports, together with task performance, suggested the manipulation was effective: participants reported elevated levels of perceived control in the 100% wins condition relative to the 100% losses condition. Furthermore, those in the 100% losses condition explored the strategy space of the game more broadly (i.e., higher strategy variability) than did those in the 100% wins condition, where presumably participants felt they had “figured out” the (non-existent) computer strategy. It is worth noting that strategy complexity was higher in the 100% wins. Had participants discovered that they would win regardless of their own play choices one would not expect to see them adopt complex strategies. In other words, it appears that participants in the 100% wins condition genuinely felt they had discovered the (illusory) exploitable strategy of their computer opponent.

Consistent with our predictions, the relationship between boredom and perceived control was best described by a U-shaped function, whereby both low *and* high perceived control were associated with elevated boredom (Figure 2). This relationship held when controlling for challenge, frustration, value, as well as measures of task engagement (i.e., strategy complexity and variability, and response time). Furthermore, no other variable demonstrated a quadratic relationship with boredom. These results suggest that both high and low levels of *perceived* control may be associated with boredom, albeit for somewhat different reasons. In both instances boredom may arise because the individual sees no point in continuing with the task. It is either too facile or too complex (Klapp, 1986; Pekrun, 2006). A high level of perceived control may promote boredom when the individual feels they have achieved complete mastery and have nothing more to achieve—the task becomes redundant. In contrast, low levels of perceived control may promote boredom when the individual feels there is little chance they could *regain* control—that is, when low levels of perceived control are seen as a barrier to engagement (i.e., the task is so noisy the participant cannot extract any meaningful signal from the noise; Klapp, 1986). In some sense, the key to boredom arising under high *or* low levels of perceived control may be the feeling that there is little *prospect* for change.

In this study, all participants were led to believe that control *could* be gained. That is, we told participants that the computer played an exploitable strategy encouraging them by their own play to discover and exploit that strategy. Further, we observed that the low perceived control condition was perceived to be less boring than the high perceived control condition. This difference may have emerged because at least some individuals in the low perceived control condition maintained the belief that they *could* discover their opponent's strategy and thereby regain some level of control over the task. In other words, boredom may have been lower in this condition because participants—erroneously—thought there was some *prospect* of gaining control. As such, any particular level of perceived control may not matter as much as the anticipation that control *could be gained*. To more specifically address this possibility, we conducted a second study in which we directly manipulated whether individuals believed that control could or could not be established.

## Experiment 2

In Experiment 2, we had participants play against an opponent that adopted a uniform random play strategy (i.e., playing each of rock, paper and scissors one third of the time). In this instance there is no play strategy that a participant can adopt that will lead to more than a 33% win rate. We assumed that this win rate would represent a condition of relatively low perceived control while allowing us to directly manipulate *perceptions* of prospective control. In the prospective control condition participants were told the computer would play an exploitable strategy. This was intended to induce the belief that participants could discover and exploit their opponent's strategy. In contrast, in the no-prospective control condition participants were told the computer played randomly. This was intended to make participants understand that their own play choices would not lead to changes in win rate.

As in Experiment 1, we included measures of state boredom, challenge, value, and frustration. We predicted that participants in the prospective control (vs. no prospective control) condition would report lower levels of boredom and demonstrate greater levels of engagement, as evidenced by our metrics of exploration of strategy space. To replicate our prior findings we also tested whether the relationship between perceived control and boredom was best described by a U-shaped function, controlling for challenge, value, and frustration.

### Method

#### Participants

Eighty-two undergraduates [58 females, mean age = 20.33 (2.38); 25.6% self-identified as East Asian, 37.8% as Caucasian, 29.3% as other Asian, and 7.3% as “other”] from the University of Waterloo, participated in exchange for course credit. Data was collected during the Fall term of 2015. It was determined, a priori, that we would collect as many participants as possible before the end of one academic term. We did not analyze data until the entire sample had been collected. This study was approved by the University of Waterloo, Office of Research Ethics and participants provided informed consent prior to participating.

#### Apparatus and Procedure

Apparatus were the same as in Experiment 1. Participants were randomly assigned to either the prospective control condition, in which participants were erroneously told the computer played an exploitable strategy, or a no-prospective control condition, in which participants were told the computer played randomly. In both conditions, participants won, lost, or tied at chance levels (i.e., each outcome had the same frequency). Thus, as with Experiment 1, the outcome of a given trial was independent of an individual's play choice. Each participant played 80 trials of RPS.

### Results

Gender proportions did not differ across conditions $X_{\left(1\right)}^{2}$ = 0.28, *p* = 0.60. No gender differences were observed across all study variables. As predicted, the no-prospective control condition was perceived to be more boring than the prospective control condition (*t* = 2.60, *p* < 0.05, *Cohen's d* = −0.55; Table 4). In addition, the prospective control condition was deemed more frustrating (*t* = 2.84, *p* < 0.01, *Cohen's d* = 0.66) and more challenging (*t* = 4.65, *p* < 0.001, *Cohen's d* = 1.01). Although ratings of perceived control were numerically higher in the prospective control condition, this difference was not significant (*t* = 1.66, *p* = 0.100, *Cohen's d* = 0.38). Perceived value was greater in the prospective control condition (*t* = 2.72, *p* < 0.01, *Cohen's d* = 0.6).

**TABLE 4:** Descriptive statistics for all variables and conditions, and independent samples comparisons, associated significance levels and effect sizes (Study 2).

		**Prospective control** ***n*** **=** **43**			**No-prospect** ***n*** **=** **39**				
**Variable**	**Mdn**	**M**	**SD**	**Mdn**	**M**	**SD**	***t***	***p***	***d***
Perceived control	26	29.7	17.81	17	22.82	19.43	1.66	0.100	0.38
Boredom	29	28.21	20.31	33	43.08	29.96	2.60	<0.05	−0.55
Frustration	31	36.58	28.56	11	20.67	22.1	2.84	<0.01	0.66
Challenge	48	41.4	27.17	5	16.44	21.31	4.65	<0.001	1.01
Value	54	52.44	23.18	29	37.1	27.42	2.72	<0.01	0.6
SC	−0.19	−0.17	0.37	−0.33	−0.38	0.26	2.38	<0.01	−0.67
SV	1.17	1.1	0.25	1.06	0.99	0.25	2.14	<0.05	0.47
RT	2.3	2.61	1.68	1.41	1.54	0.49	3.96	<0.001	0.88

*Mdn, median; M, mean; SD, standard deviation; d, Cohen's d. Abbreviations as for Table 2*.

Zero-order correlations between all study variables showed that boredom was negatively associated with value (*r* = −0.29, *p* < 0.01) and response time (*r* = −0.24, *p* < 0.05). Perceived control was positively associated with strategy complexity (*r* = 0.23, *p* < 0.001). There was no association between perceived control and value (*r* = 0.14, *p* = 0.20; Table 5A shows correlations collapsed across conditions; Table 5B shows correlations for the *Prospective Control* condition; Table 5C shows correlations for the *No Prospective Control* condition).

**TABLE 5A:** Zero-order correlations of all study variables and their significance levels (Study 2).

	**1**	**2**	**3**	**4**	**5**	**6**	**7**
1. Perceived control							
2. Bored	0.02						
3. Frustration	−0.08	−0.1					
4. Challenge	0.07	−0.13	0.51^***^				
5. Value	0.14	−0.29^**^	0.4^***^	0.32^**^			
6. Strategy complexity	0.23^*^	−0.01	0.17	0.13	0.1		
7. Strategy variability	0.18	0.04	0.2	0.15	0.07	0.86^***^	
8. Response time	−0.01	−0.24^*^	0.39^***^	0.46^***^	0.34^**^	0.32^**^	0.23^*^

* *p < 0.05*,

** *p < 0.01*,

*** *p < 0.001*.

**TABLE 5B:** Zero-order correlations of all study variables and their significance levels (Study 2: Prospective Control).

	**1**	**2**	**3**	**4**	**5**	**6**	**7**
1. Perceived control							
2. Bored	0.15						
3. Frustration	−0.24	0.24					
4. Challenge	−0.2	0.03	0.55^***^				
5. Value	−0.08	−0.32^*^	0.42^**^	0.37^*^			
6. Strategy complexity	0.32^*^	0.08	0.06	−0.09	0.05		
7. Strategy variability	0.25	0.16	0.08	0.03	0.02	−0.84^***^	
8. Response time	−0.09	−0.28	0.37^*^	0.35^*^	0.39^**^	−0.23	0.17

* *p < 0.05*,

** *p < 0.01*,

*** *p < 0.001*.

**TABLE 5C:** Zero-order correlations of all study variables and their significance levels (Study 2: No-Prospect Control).

	**1**	**2**	**3**	**4**	**5**	**6**	**7**
1. Perceived control							
2. Bored	0.03						
3. Frustration	−0.02	−0.26					
4. Challenge	0.21	−0.04	0.23				
5. Value	0.25	−0.16	0.26	0.06			
6. Strategy complexity	−0.01	0.1	0.13	0.14	−0.06		
7. Strategy variability	0.03	0.08	0.23	0.07	−0.01	−0.90^***^	
8. Response time	−0.15	0.08	0.1	0.46^**^	0.04	−0.24	0.16

** *p < 0.01*,

*** *p < 0.001*.

**Figure 4:**
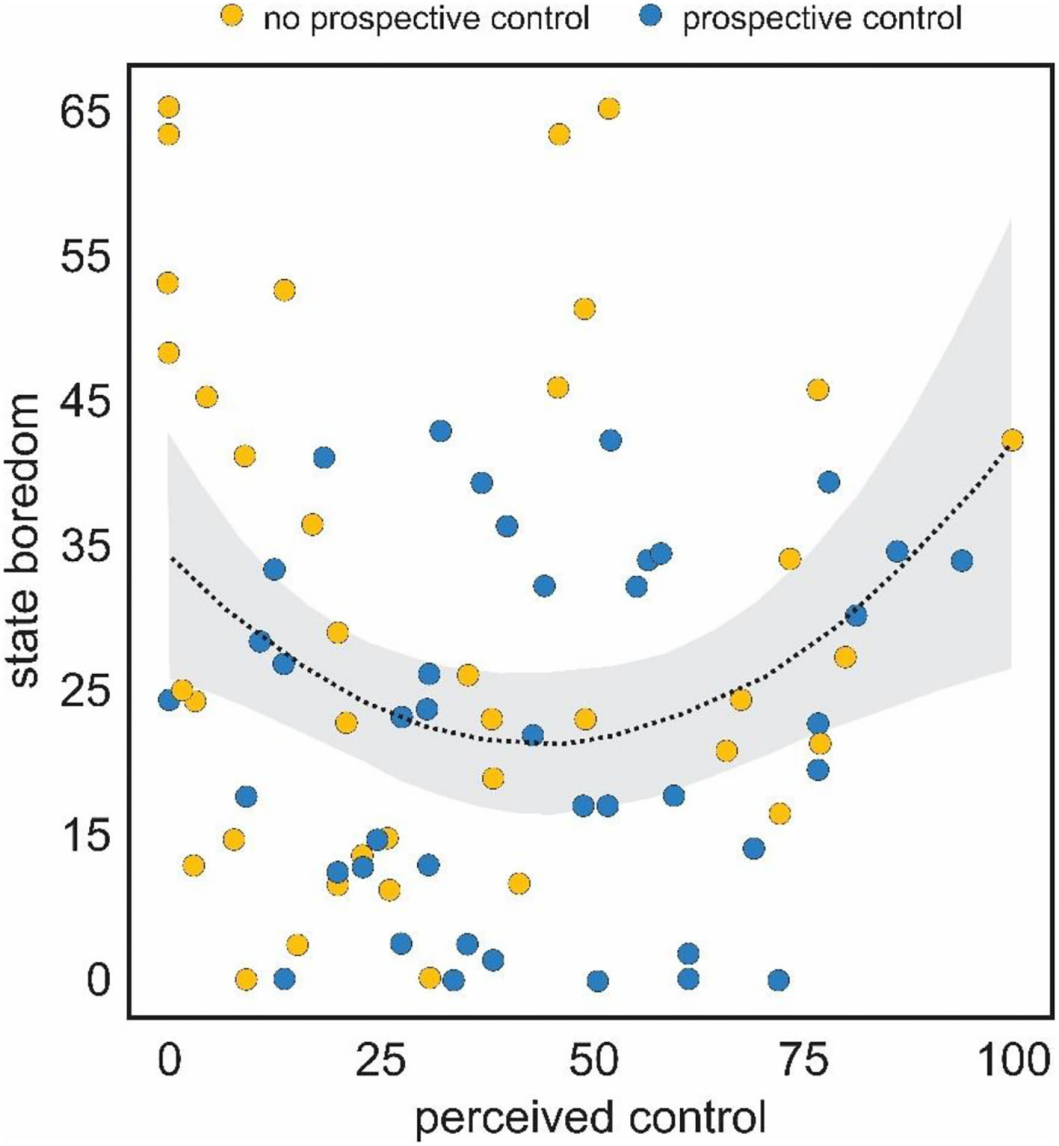
Curvilinear relationship between self-reported ratings of state boredom (y-axis) and perceived control (x-axis). Boredom was highest at the low and high ends of perceived control.

Next, we examined whether perceived control demonstrated a quadratic relationship with boredom. All variables were scaled and centered and a new squared variable generated and we tested whether the second order polynomial of perceived control incrementally improved the model fit. The linear relationship between perceived control and boredom was non-significant [*F*_(1,80)_ = 0.032, *p* = 0.858, adjusted *R*^2^ = −0.012]. Addition of the quadratic term significantly improved the model fit [*F*_(1,79)_ = 4.36, *SS* = 4.23, *p* = 0.04]. The linear effect was non-significant (β = −0.10, *p* = 0.42), but the quadratic term was a significant (β = 0.26, *p* = 0.045) predictor of boredom. The overall model fit was non-significant [*F*_(2,79)_ = 2.195, *p* = 0.118, adjusted *R*^2^ = 0.0286]. Although not as robust as the results of Experiment 1, these results corroborate our prior findings, suggesting the relationship between perceived control and boredom is best described as quadratic (Figure 4). Note that no other variable, including challenge, demonstrated a curvilinear relationship with boredom.

**Figure 5:**
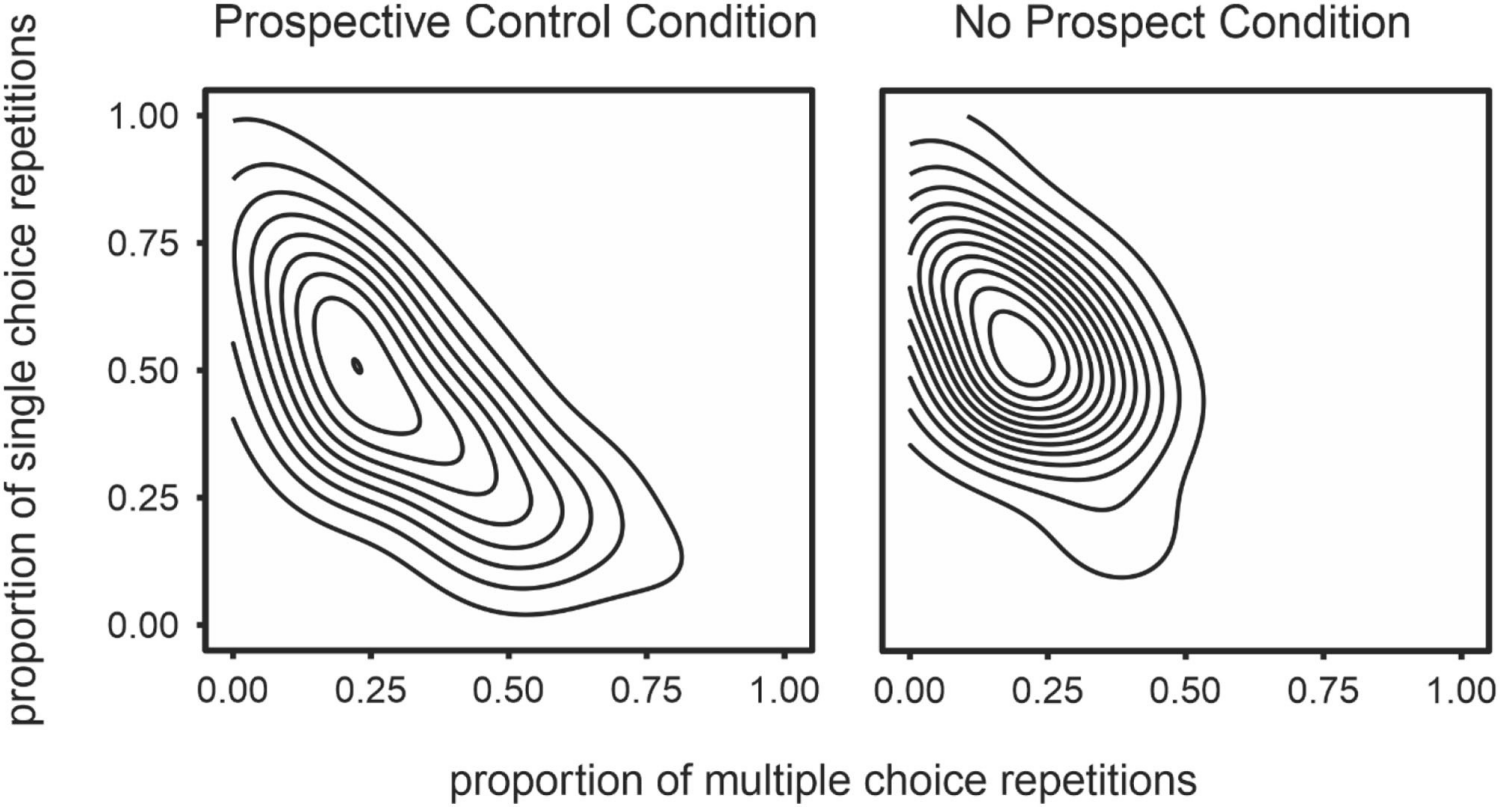
Density plots representing exploration of strategy space in the prospective control **(left)** and no prospective control **(right)** conditions. The proportion of trials on which play involved complex strategies (alternations and sequences) are presented on the x-axis and the proportion of trials where a single choice was played repeatedly is presented on the y-axis. The density plot was generated by collapsing across all participants, for each condition separately. Regions with many concentric lines indicate a high density or frequency of that particular distribution of strategies.

The prospective control condition was characterized by high strategy variability (*t* = 2.14, *p* < 0.05, *Cohen's d* = 0.47; Figure 5) and long response times (*t* = 3.96, *p* < 0.001, *Cohen's d* = 0.88). In contrast, the no-prospect condition was associated with a preference toward simple strategies (*t* = 2.38, *p* < 0.01, *Cohen's d* = −0.67; Figure 5). That is, although the modal density for each condition was similar, participants in the no-prospect condition adopted a single response strategy more prominently (Figure 5).

To explore whether perceived value independently predicts boredom beyond perceived control, we ran a hierarchical regression analysis. Value was added as a predictor to a model that had the linear and quadratic terms of perceived control as predictors of boredom. Adding value significantly improved the fit [*F*_(1,78)_ = 5.43, *SS* = 5.00, *p* < 0.05], accounting for an additional 5.1% of variance (comparable to Experiment 1). Value was a significant negative predictor (β = −0.225, *p* < 0.05) of boredom and the linear term of perceived control continued to operate as a non-significant negative predictor (β = −0.030, *p* = 0.807). The quadratic term now operated as a non-significant positive predictor (β = 0.19, *p* = 0.129), with an overall significant model fit [*F*_(3,78)_ = 3.36, *p* < 0.05, adjusted *R*^2^ = 0.080].

### Discussion

In Experiment 2, we directly examined how the belief that control could be gained affected ratings of boredom. To do this, we manipulated whether participants had a sense of prospective control by telling them the computer played an exploitable strategy or not. Even though all participants experienced the same low win rate, the mere belief that there was something to exploit led those participants to experience higher levels of perceived control. Consistent with our hypothesis, the prospective control condition was seen as less boring, more engaging, more valuable, *and* more frustrating. These findings suggest that an individual's beliefs about prospects of gaining control provide a buffer against boredom, wherein individuals find it to be an opportunity to satisfy the need for control and are more inclined to stay engaged. Notably, this was true even though the game play for Experiment 2 spanned many more trials than Experiment 1, suggesting that time on task did not diminish the effects induced by the belief that control could be gained. These findings also help construct a more general case that when individuals believe control *cannot* be gained, they will abandon attempts to establish control, and thus remain unengaged and bored.

## General Discussion

The purpose of this research was to investigate the relationship between perceived control and in-the-moment feelings of boredom. As predicted, we observed a quadratic relationship such that individuals reported more boredom at both low and high levels of perceived control. It should be pointed out that each end of this quadratic indicates something distinct about the relation. At low levels of perceived control the relation is negative—as levels of perceived control rise slightly, boredom diminishes. At high levels of perceived control the relation is positive—as levels of perceived control rise so too do boredom levels. These distinct relations hint at distinct mechanisms. On the one hand (low perceived control) boredom may reflect a feeling that we have no chance of influencing outcomes—a loss of the sense of agency. Whatever we choose to do is pointless as the outcomes won't change. On the other (high perceived control), we may believe that our mastery indicates that there is nothing new to learn—the task (and our interaction with it) have become redundant (Klapp, 1986). The results of our second experiment suggested that whether situations of low perceived control result in boredom depends less on absolute levels of perceived control, and more on perceived *opportunities (or lack thereof)* of gaining control. It is perhaps worth noting here that we did not ask participants in either experiment whether or not they were aware of our manipulation. We did ask, however, how important winning was to them with mean ratings falling in the middle of our scale for both experiments (value ratings in Tables 3, 4 which were made in response to the question “How much do you care about winning?”—see Table 1). Had participants discovered our ruse one would expect their value ratings to drop accordingly.

Our results provide evidence that perception of control plays an important role in the experience of boredom. Whether and how these experiences generalize to other contexts will be important to explore in future work. While we were able to manipulate the outcomes in the rock-paper-scissors game used here, the task itself has some idiosyncratic features. The paradigm requires a simple repeated response, which makes it difficult to generalize to the role of perceived control on boredom and task engagement during more complex goal pursuit. Additionally, it is possible that participants had lay beliefs about the role of chance and skill that influenced how the manipulations affected perceptions of control. Future studies using diverse tasks and manipulations of perceived (or actual) control will help to better understand the dynamics of the relationship between control and boredom.

In addition, our manipulation of perceived control may have also had an influence on feelings of agency. That is, when we perceive a high level of control we may simultaneously feel like we are effectively exerting agency over the task at hand. It has been suggested that the inability to engage in a satisfying activity brought on by boredom, may be perceived as a threat to one's sense of agency (see Kahn, 2018 for preliminary evidence that boredom is associated with a need to establish agency). Future work could explore this relation more directly by manipulating agency independent of perceived control.

An additional limitation might be assumed from the absence of a “middle” level of manipulated perceptions of control (or indeed a manipulation intended to induce optimal feelings of control, challenge etc.). We decided against attempting to create such a condition for several reasons. First, in our initial uses of this task (Danckert et al., 2012) healthy individuals found it challenging to exploit a 50% bias in play (i.e., rock played 50% of the time by the computer opponent) and found it facile to exploit an 80% bias (e.g., paper played 80% of the time). It was not clear then, what an optimal challenge point would be. Nevertheless, future work could attempt to titrate a task to achieve just such a point. Second, we were attempting to induce feelings of boredom at both a high and low end of perceived control. A median control manipulation would not be expected to induce boredom. Finally, perhaps fortuitously, our manipulation did successfully lead to a broad range of perceived control ratings (Figures 2, 4) allowing for a robust examination of the hypothesized quadratic relation between boredom and perceptions of control.

Given that judgments concerning prospects of control are subjective, individuals need to constantly evaluate those prospects relative to the outcomes of actions. Continuing attempts at gaining control in the face of failure represent banging one's head against a wall. Discontinuing efforts to establish control in such circumstances represents an adaptive course of action. In our experiments participants are not availed of that course of action and so often experienced elevated levels of frustration. Presumably, given enough time, boredom follows such frustrated attempts to engage. Thus, frustration and boredom, although distinct states, are temporally related and may appear to co-vary when considering a longer time span, such as retrospective probes following a classroom setting, or when considering trait boredom. In these cases individuals are more likely to become *both* frustrated *and* bored, since both states stem from a failure to establish a sense of control.

Consistently, in studies that have assessed longer timespans (Dicintio and Gee, 1999; Pekrun et al., 2010), low perceived control has been associated with high levels of boredom. This is likely because in these situations individuals who had low perceived control also had diminished *prospects* of gaining control. This is in contrast to our current findings in which individuals reporting a low sense of perceived control within a circumstance in which there remained a prospect of gaining control were not as bored. Future research may benefit from investigating how boredom evolves from frustrated engagement over time to test the mediating role of diminishing prospects of gaining control. In addition, monotony likely plays a key role (certainly, monotony is a strong driver of boredom; Thackray, 1981). In the experiments presented here we did not directly measure perceptions of monotony. Whatever influence it may have had ought to have been equivalent across all of our manipulations. In Experiment 1 the participant either *always* wins or *always* loses—either way, the outcomes are consistent and monotonous. In Experiment 2, in both conditions the participant is only capable of *winning at chance levels*. So, while play from trial to trial may change, outcomes in a more general sense are equivalently monotonous in both conditions. It is possible that for participants in the “no prospective control” condition that this was *felt* to be more monotonous, something that future work ought to explore more directly.

There are some clear limitations to this work. First, we have cast our manipulations as influencing one's perceived sense of control. While the data clearly show that only perceived control shows a quadratic relation to boredom, there were also clear differences in our conditions in perceived challenge and value. We cannot rule out that these factors played an important role. Indeed, a great deal of boredom research casts the state feeling as driven by a felt lack of value, purpose or meaning related to the task at hand (e.g., Pekrun, 2006; van Tilburg and Igou, 2011). It is likely that low challenging tasks—winning at rock, paper, scissors all the time—are experienced as lacking in meaning and are thus felt as boring. Rather than suggesting that researchers try to isolate characteristics like control, challenge and value, we would suggest that these factors are inextricably linked. To attempt to experimentally isolate a single factor may be like building a ship in a bottle—every move you make brings you up against a constraint. Pitting challenge and control against one another is difficult. If a task is kept to a low challenge level it may be perceived as meaningless and boring, negating any impact of a manipulation of control. If a task is kept to a uniformly high level of challenge, all participants may feel they are unable to attain sufficient control to succeed. Finally, defining an “optimal” level of challenge on a per participant basis would necessitate some feedback indicative of successful task performance, sacrificing any manipulation of control (i.e., succeeding at the task would necessarily imply you are in control of what is happening).

Westgate and Wilson (2018) proposed a model of boredom that explicitly carves the feeling state at the joints of attentional control and meaning. A hybrid of other models of boredom that focus on attention (Eastwood et al., 2012) or meaning (van Tilburg and Igou, 2011; Tam et al., 2021), they highlight the notion that boredom as a feeling state is influenced by a broad range of contingencies (e.g., should we include skill-challenge fit in the Westgate and Wilson model?) that likely interact to produce the experience. We may more profitably understand those contingencies by allowing them to freely vary experimentally and finding sensitive metrics to gage dynamic changes in boredom (e.g., Allen et al., 2016).

In addition to the difficulty of isolating a single antecedent to boredom, we have also manipulated the extreme ends of the domain of interest. That is, we engaged two conditions intend to produce high (100% wins) or low (100% losses) levels of perceived control, with no explicit manipulation of moderate levels of control. Despite our dichotomous task conditions, the *reported* levels of perceived control spanned the full range of possible reports—from 0, an admission of feeling absolutely no sense of control, to 100, a claim that indicates one feels completely in control of the task (Figures 2, 4). As such, the wide range of perceptions of control were sufficient for us to test our hypothesis that the relation between boredom and perceived control would be quadratic, which it was (Figures 2, 4).

We could also cast our findings not in terms of perceived control, but in terms of information gain (or lack thereof; Klapp, 1986). In a clever study exploring this possibility, Geana et al. (2016) had participants perform a number prediction task under three conditions: one in which the numbers generated by the computer were drawn from a Gaussian distribution and as such could be learned by the participant, a second condition in which the distribution was uniform and random, and a third condition in which the number to be predicted was known to the participant on all trials (i.e., this amounted to having participants type in a number displayed on the screen—a completely facile task). They found that the completely predictable condition was rated to be most boring, while the Gaussian condition, in which information could be learned and profitably used to improve performance, was the least boring. The parallels here are between the uniform distribution and our 100% loss condition (in both the participant's choices are irrelevant), and between the 100% wins condition and the completely predictable number generation task (i.e., in both the task is facile, although more obviously so in Geana's study). Cast as information gain, there is no new information to be gleaned from either situation. Either the number you generate or rock, paper, scissors choice you make will be right by chance (100% losses, uniform number generation), or choices made are always right (100% wins and completely predictable number generation; Geana et al., 2016). We cannot disentangle from our current data sets whether the perceived sense of control was driven by representations of information gain—or more precisely, the lack thereof. This framing of boredom casts it in terms of opportunity costs (Kurzban et al., 2013; Struk, 2020) which is an intriguing possibility.

An individual's perceived sense of autonomy, agency and control are likely key drivers of boredom. Over the past year, restrictions imposed as a function of the COVID-19 pandemic have seriously curtailed autonomy over one's actions. Research has shown that the highly boredom prone tend to break the rules of social distancing, perhaps in response to this constraint on autonomy (Wolff et al., 2020; Boylan et al., 2021). The work presented here highlights the important role beliefs of control have on our tendency to engage in tasks and experience in-the-moment feelings of boredom. Existing theories emphasize discrepancies between skill and task demands as factors leading to boredom, suggesting boredom may be targeted by providing opportunities appropriate to a given skill level (Csikszentmihalyi, 1975; Troutwine and O'Neal, 1981; Dicintio and Gee, 1999; Moneta and Csikszentmihalyi, 1999; Watt and Vodanovich, 1999; Kanevsky and Keighley, 2003; Pekrun, 2006; van Tilburg and Igou, 2012). The current research suggests that perceptions of control, not simply challenge, play a key role in boredom, perhaps by influencing how individuals maintain and initiate task engagement, highlighting the importance of investigating affective regulation strategies in boredom interventions.

## Data Availability Statement

The raw data supporting the conclusions of this article will be made available by the authors, without undue reservation.

## Ethics Statement

The studies involving human participants were reviewed and approved by University of Waterloo's Office of Research Ethics. The patients/participants provided their written informed consent to participate in this study.

## Author Contributions

ASt collected the data, conducted primary analyses, and wrote the first draft. ASc and JD contributed to redrafting. All authors contributed equally to task design and secondary analyses.

## Conflict of Interest

The authors declare that the research was conducted in the absence of any commercial or financial relationships that could be construed as a potential conflict of interest.
